# Analysis of SLICK allele in African taurine and Zebu cattle breeds

**DOI:** 10.1111/age.13499

**Published:** 2024-12-22

**Authors:** Samrawit Gebeyehu, Bradley Heins, Tad Sonstegard, Johann Sölkner, Gábor Mészáros, Amadou Traoré, Albert Soudré

**Affiliations:** ^1^ Department of Animal Science University of Minnesota St Paul Minnesota USA; ^2^ Acceligen Inc. Eagan Minnesota USA; ^3^ Department of Sustainable Agricultural Systems University of Natural Resources and Life Sciences Vienna (BOKU) Vienna Austria; ^4^ Institut de l'Environnement et de Recherches Agricoles (INERA) Ouagadougou Burkina Faso; ^5^ Unité de Formation et de Recherche en Sciences et Technologies Université Norbert Zongo Koudougou Burkina Faso

The SLICK trait enhances heat tolerance by modifying physiological traits, such as improving sweating ability, and is phenotypically expressed as short and sleek coats (Davis et al., 2016; Olson et al., 2003). The SLICK coat trait in cattle is attributed to six naturally occurring truncation mutations in the prolactin receptor (PRLR) gene on bovine chromosome 20 (Flórez et al., 2020; Sosa et al., 2021). This results in frameshift mutations in the PRLR gene, particularly in exons 10 and 11, which disrupt the open reading frame, and leads to truncated receptor proteins that confer the SLICK phenotype (Davis et al., 2017; Porto‐Neto et al., 2018). These mutations have been identified in many South American breeds, such as Senepol, Carora and Limonero, and the trait is inherited in an autosomal‐dominant manner (Flórez et al., 2020; Flórez Murillo et al., 2024; Nicholas et al., 2024; Olson et al., 2003). The mutation was first identified in Criollo breeds: the Mexican Criollo Lechero has SLICK3 and SLICK4 at frequencies of 0.9 and 0.03; the Colombian Blanco Orejineg has SLICK1 and SLICK5 at frequencies of 0.50 and 0.38; and the Hartón del Valle has SLICK1, SLICK2, SLICK5 and SLICK6 at frequencies of 0.29, 0.18, 0.24 and 0.21 respectively (Flórez et al., 2020). Although the allelic frequency of the mutation is relatively low in some Criollo breeds, the autosomal‐dominant inheritance pattern ensures that individuals carrying at least one copy of a SLICK allele exhibit the phenotype.

The SLICK allele is well documented in Criollo breeds of the Caribbean Basin, descended from Iberian taurine cattle (Flórez et al., 2020; Porto‐Neto et al., 2018). The allele may also exist in African breeds because these cattle have environmental stressors similar to those of Criollo breeds. However, the specific SLICK mutation in African taurine cattle has not been identified (Xia et al., 2023). Although African cattle possess various tropical adaptations, it is unknown whether they exhibit the SLICK phenotype. Investigation of the presence and frequency of SLICK alleles in West African breeds is crucial for understanding the origins of these mutations and their potential application in breeding programs to enhance adaptability, productivity and resilience in tropical dairy systems. The study evaluated SLICK‐causing mutations in African breeds compared with Criollo breeds. No SLICK mutations were found in a panel of 1063 genomic DNA samples from 40 breeds across 15 ecotypes, including 126 African taurine samples (Sonstegard et al., 2025). Genotyping of SLICK mutations (1–5) was done with iPlex assays (Geneseek, Lincoln, NE, USA). Allele frequencies were determined for four Criollo breeds (Mexican Criollo Lechero tropical, *n* = 20; Colombian Blanco Orejinegro, *n* = 40; Hartón del Valle, *n* = 71; Caracu, *n* = 29), four Zebu breeds (Brahman, *n* = 5; Bunaji, *n* = 30; Gir, *n* = 245; West African Zebucross, *n* = 50), two West African taurine breeds (Muturu, *n* = 30; Baoulé, *n* = 96), and one Sanga breed (Mashona, *n* = 37).

The genotypic analysis found the presence of the SLICK2 variant in West African taurine and Zebu crossbred cattle (Table 1). This study is the first to report the presence of SLICK2 alleles in West African cattle, which makes it a novel finding that expands our understanding of cattle genetics in Africa. Despite the relatively low allelic frequency of the mutation, the autosomal‐dominant inheritance pattern ensures that individuals carrying at least one copy of a SLICK allele exhibit the phenotype. The discovery of SLICK2 alleles in Burkina Faso suggests two possible scenarios for the variant: it originated in Africa or was brought to Africa from South America during colonial trading times. The prevalence of SLICK alleles is highest in Criollo breeds, which are known carriers of multiple heat‐tolerance variants. Notably, the HV breed uniquely carries both SLICK1 and SLICK5, independent genetic variations contributing to the same phenotype (Porto‐Neto et al., 2018). The finding underscores the potential value of incorporating SLICK alleles into African breeding programs, offering a promising avenue for enhancing thermotolerance and productivity in tropical systems. This could significantly improve the resilience and adaptability of African cattle, providing a clear motivation for further research and application.

**TABLE 1:** Allelic frequency of SLICK variants of the prolactin receptor (PRLR) gene in Criollo, West African taurine and Zebu cattle.

Allele name	Reference allele and variant		Breed category										
			Criollo breed				West African taurine		Zebu				Sanga
			CLT	BON	HV	Ca	Ba	Mu	Br	Bu	Gir	Za	Ma
SLICK1	Mutant	Cdel	0	0	0.32	0	0	0	0	0	0	0	0
	Wildtype	C	1	1	0.68	1	1	1	1	1	1	1	1
SLICK2	Mutant	T	0	0.025	0	0.89	0.01	0	0	0	0	0.02	0
	Wildtype	C	1	0.075	0	0.1	0.99	1	1	1	1	0.98	1
SLICK3	Mutant	A	1	0	0	0	0	0	0	0	0	0	0
	Wildtype	C	0	1	1	1	1	1	1	1	1	1	1
SLICK4	Mutant	GC	0.05	0	0	0	0	0	0	0	0	0	0
	Wildtype	C	0.95	1	1	1	1	1	1	1	1	1	1
SLICK5	Mutant	T	0	0	0.89	0	0	0	0	0	0	0	0
	Wildtype	A	1	1	0.11	1	1	1	1	1	1	1	1

Abbreviations: Ba, Baoulé; BON, Colombian Blanco Orejinegro; Br, Brahman; Bu, Bunaji; Ca, Caracu; CLT, Mexican Criollo Lechero tropical; Gir, Indian Zebu cattle; HV, Hartón del Valle cattle; Ma, Mashona; Mu, Muturu; Za, West African Zebucross breeds.

## AUTHOR CONTRIBUTIONS

**Samrawit Gebeyehu:** Investigation; writing – original draft; validation; visualization; writing – review and editing; data curation; software; resources; formal analysis; methodology. **Bradley Heins:** Investigation; writing – review and editing; validation; visualization; software; supervision; resources; formal analysis; methodology. **Tad Sonstegard:** Conceptualization; investigation; funding acquisition; validation; writing – review and editing; project administration; formal analysis; methodology; data curation; resources. **Johann Sölkner:** Investigation; writing – review and editing; resources. **Gábor Mészáros:** Resources; writing – review and editing; investigation. **Amadou Traoré:** Investigation; writing – review and editing; resources. **Albert Soudré:** Investigation; writing – review and editing; resources.

## FUNDING INFORMATION

This study was funded by the Bill and Melinda Gates Foundation (INV‐004986).

## CONFLICT OF INTEREST STATEMENT

Tad Sonstegard is an employee of Recombinetics. Recombinetics is licensed to use DNA markers to determine SLICK alleles.

## Supporting information


Table S1.

## Data Availability

The iPlex assay designs and SNP genotyping data are available upon request from the corresponding author.
