# Supplementary material for: Analysis of SLICK allele in African taurine and Zebu cattle breeds
Source: Anim Genet. 2024 Dec 22;56(1):e13499. doi: 10.1111/age.13499 (PMC11664028; doi:10.1111/age.13499)
Supplement: Supplementary file 1 — Table S1. [file AGE-56-0-s001.docx]

**Supporting information**

**Table S1**: Genotypic frequency of the PRLR mutations in Criollo, West African Taurine, and Zebu cattle breeds

| \| **Allele name** \| **Genotype** \|  \|  \|  \|  \| **Breed** \|  \|  \|  \|  \|  \|  \| \| \| --- \| --- \| --- \| --- \| --- \| --- \| --- \| --- \| --- \| --- \| --- \| --- \| --- \| --- \| \| **CLT** \| **BON** \| **HV** \| **Ca** \| **Mu** \| **Br** \| **Ba** \| **Ma** \| **Bu** \| **Gir** \| \| **ZC** \| \| **SLICK1** \| +/+ \| 0 \| 0 \| 0.29 \| 0 \| 0 \| 0 \| 0 \| 0 \| 0 \| 0 \| \| 0 \| \| +/- \| 0 \| 0 \| 0.029 \| 0 \| 0 \| 0 \| 0 \| 0 \| 0 \| 0 \| \| 0 \| \| -/- \| 1 \| 1 \| 0.68 \| 1 \| 1 \| 1 \| 1 \| 1 \| 1 \| 1 \| \| 1 \| \| **SLICK2** \| +/+ \| 0 \| 0 \| 0 \| 0.55 \| 0 \| 0 \| 0 \| 0 \| 0 \| 0 \| \| 0 \| \| +/- \| 0 \| 0.025 \| 0 \| 0.34 \| 0 \| 0 \| 0.01 \| 0 \| 0 \| 0 \| \| 0.02 \| \| -/- \| 1 \| 0.075 \| 0 \| 0.1 \| 1 \| 1 \| 0.99 \| 1 \| 1 \| 1 \| \| 0.98 \| \| **SLICK3** \| +/+ \| 0.8 \| 0 \| 0 \| 0 \| 0 \| 0 \| 0 \| 0 \| 0 \| 0 \| \| 0 \| \| +/- \| 0.2 \| 0 \| 0 \| 0 \| 0 \| 0 \| 0 \| 0 \| 0 \| 0 \| \| 0 \| \| -/- \| 0 \| 1 \| 1 \| 1 \| 1 \| 1 \| 1 \| 1 \| 1 \| 1 \| \| 1 \| \| **SLICK4** \| +/+ \| 0 \| 0 \| 0 \| 0 \| 0 \| 0 \| 0 \| 0 \| 0 \| 0 \| \| 0 \| \| +/- \| 0.05 \| 0 \| 0 \| 0 \| 0 \| 0 \| 0 \| 0 \| 0 \| 0 \| \| 0 \| \| -/- \| 0.95 \| 1 \| 1 \| 1 \| 1 \| 1 \| 1 \| 1 \| 1 \| 1 \| \| 1 \| \| **SLICK5** \| +/+ \| 0 \| 0 \| 0.59 \| 0 \| 0 \| 0 \| 0 \| 0 \| 0 \| 0 \| \| 0 \| \| +/- \| 0 \| 0 \| 0.3 \| 0 \| 0 \| 0 \| 0 \| 0 \| 0 \| 0 \| \| 0 \| \| -/- \| 1 \| 1 \| 0.11 \| 1 \| 1 \| 1 \| 1 \| 1 \| 1 \| 1 \| \| 1 \|   A genotype (+/+) indicates the presence of the SLICK variant as a homozygote, +/- indicates the SLICK variant as being heterozygote, and -/- means the absence of the SLICK variant (indicates wildtype). CLT: Mexican Criollo Lechero tropical; HV: Hartón del Valle cattle; BON: Colombian Blanco Orejinegro; Ca: Caracu; Mu: Muturu; Br: Brahman; Ba: Baoulé, Ma: Mashona; Bu: Bunaji; ZC: West African Zebu cross breeds |
| --- | --- | --- | --- | --- | --- | --- | --- | --- | --- | --- | --- | --- | --- | --- | --- | --- | --- | --- | --- | --- | --- | --- | --- | --- | --- | --- | --- | --- | --- | --- | --- | --- | --- | --- | --- | --- | --- | --- | --- | --- | --- | --- | --- | --- | --- | --- | --- | --- | --- | --- | --- | --- | --- | --- | --- | --- | --- | --- | --- | --- | --- | --- | --- | --- | --- | --- | --- | --- | --- | --- | --- | --- | --- | --- | --- | --- | --- | --- | --- | --- | --- | --- | --- | --- | --- | --- | --- | --- | --- | --- | --- | --- | --- | --- | --- | --- | --- | --- | --- | --- | --- | --- | --- | --- | --- | --- | --- | --- | --- | --- | --- | --- | --- | --- | --- | --- | --- | --- | --- | --- | --- | --- | --- | --- | --- | --- | --- | --- | --- | --- | --- | --- | --- | --- | --- | --- | --- | --- | --- | --- | --- | --- | --- | --- | --- | --- | --- | --- | --- | --- | --- | --- | --- | --- | --- | --- | --- | --- | --- | --- | --- | --- | --- | --- | --- | --- | --- | --- | --- | --- | --- | --- | --- | --- | --- | --- | --- | --- | --- | --- | --- | --- | --- | --- | --- | --- | --- | --- | --- | --- | --- | --- | --- | --- | --- | --- | --- | --- | --- | --- | --- | --- | --- | --- | --- | --- | --- | --- | --- | --- | --- | --- | --- | --- | --- | --- | --- | --- | --- | --- | --- | --- | --- | --- | --- | --- |

| \| Allele name \| **Genotype** \|  \|  \|  \|  \| \| **Breed** \| \|  \|  \|  \|  \|  \|  \| \| --- \| --- \| --- \| --- \| --- \| --- \| --- \| --- \| --- \| --- \| --- \| --- \| --- \| --- \| --- \| \| **CLT** \| **BON** \| **HV** \| **Ca** \| \| **Mu** \| **Br** \| \| **Ba** \| **Ma** \| **Bu** \| **Gir** \| **ZC** \| \| SLICK1 \| +/+ \| 0 \| 0 \| 0.29 \| 0 \| \| 0 \| 0 \| \| 0 \| 0 \| 0 \| 0 \| 0 \| \| +/- \| 0 \| 0 \| 0.029 \| \| 0 \| 0 \| 0 \| \| 0 \| 0 \| 0 \| 0 \| 0 \| \| -/- \| 1 \| 1 \| 0.68 \| 1 \| \| 1 \| 1 \| \| 1 \| 1 \| 1 \| 1 \| 1 \| \| SLICK2 \| +/+ \| 0 \| 0 \| 0 \| 0.55 \| \| 0 \| 0 \| \| 0 \| 0 \| 0 \| 0 \| 0 \| \| +/- \| 0 \| 0.025 \| 0 \| 0.34 \| \| 0 \| 0 \| \| 0.01 \| 0 \| 0 \| 0 \| 0.02 \| \| -/- \| 1 \| 0.075 \| 0 \| 0.1 \| \| 1 \| 1 \| \| 0.99 \| 1 \| 1 \| 1 \| 0.98 \| \| SLICK3 \| +/+ \| 0.8 \| 0 \| 0 \| 0 \| \| 0 \| 0 \| \| 0 \| 0 \| 0 \| 0 \| 0 \| \| +/- \| 0.2 \| 0 \| 0 \| 0 \| \| 0 \| 0 \| \| 0 \| 0 \| 0 \| 0 \| 0 \| \| -/- \| 0 \| 1 \| 1 \| 1 \| \| 1 \| 1 \| \| 1 \| 1 \| 1 \| 1 \| 1 \| \| SLICK4 \| +/+ \| 0 \| 0 \| 0 \| 0 \| \| 0 \| 0 \| \| 0 \| 0 \| 0 \| 0 \| 0 \| \| +/- \| 0.05 \| 0 \| 0 \| 0 \| \| 0 \| 0 \| \| 0 \| 0 \| 0 \| 0 \| 0 \| \| -/- \| 0.95 \| 1 \| 1 \| 1 \| \| 1 \| 1 \| \| 1 \| 1 \| 1 \| 1 \| 1 \| \| SLICK5 \| +/+ \| 0 \| 0 \| 0.59 \| 0 \| \| 0 \| 0 \| \| 0 \| 0 \| 0 \| 0 \| 0 \| \| +/- \| 0 \| 0 \| 0.3 \| 0 \| \| 0 \| 0 \| \| 0 \| 0 \| 0 \| 0 \| 0 \| \| -/- \| 1 \| 1 \| 0.11 \| 1 \| \| 1 \| 1 \| \| 1 \| 1 \| 1 \| 1 \| 1 \|   A genotype (+/+) indicates the presence of the SLICK variant as a homozygote, +/- indicates the SLICK variant as being heterozygote, and -/- means the absence of the SLICK variant (indicates wildtype). CLT: Mexican Criollo Lechero tropical; HV: Hartón del Valle cattle; BON: Colombian Blanco Orejinegro; Ca: Caracu; Mu: Muturu; Br: Brahman; Ba: Baoulé, Ma: Mashona; Bu: Bunaji; ZC: West African Zebu cross breeds |
| --- | --- | --- | --- | --- | --- | --- | --- | --- | --- | --- | --- | --- | --- | --- | --- | --- | --- | --- | --- | --- | --- | --- | --- | --- | --- | --- | --- | --- | --- | --- | --- | --- | --- | --- | --- | --- | --- | --- | --- | --- | --- | --- | --- | --- | --- | --- | --- | --- | --- | --- | --- | --- | --- | --- | --- | --- | --- | --- | --- | --- | --- | --- | --- | --- | --- | --- | --- | --- | --- | --- | --- | --- | --- | --- | --- | --- | --- | --- | --- | --- | --- | --- | --- | --- | --- | --- | --- | --- | --- | --- | --- | --- | --- | --- | --- | --- | --- | --- | --- | --- | --- | --- | --- | --- | --- | --- | --- | --- | --- | --- | --- | --- | --- | --- | --- | --- | --- | --- | --- | --- | --- | --- | --- | --- | --- | --- | --- | --- | --- | --- | --- | --- | --- | --- | --- | --- | --- | --- | --- | --- | --- | --- | --- | --- | --- | --- | --- | --- | --- | --- | --- | --- | --- | --- | --- | --- | --- | --- | --- | --- | --- | --- | --- | --- | --- | --- | --- | --- | --- | --- | --- | --- | --- | --- | --- | --- | --- | --- | --- | --- | --- | --- | --- | --- | --- | --- | --- | --- | --- | --- | --- | --- | --- | --- | --- | --- | --- | --- | --- | --- | --- | --- | --- | --- | --- | --- | --- | --- | --- | --- | --- | --- | --- | --- | --- | --- | --- | --- | --- | --- | --- | --- | --- | --- | --- | --- | --- | --- | --- | --- | --- | --- | --- | --- | --- | --- | --- | --- | --- | --- | --- | --- | --- |
